# MMP-1 promoter polymorphism is associated with risk of radiation-induced lung injury in lung cancer patients treated with radiotherapy

**DOI:** 10.18632/oncotarget.12164

**Published:** 2016-09-21

**Authors:** Bo Liu, Minxiao Yi, Yang Tang, Qingxu Liu, Hong Qiu, Yanmei Zou, Ping Peng, Lu Zhang, Chunping Hu, Xianglin Yuan

**Affiliations:** ^1^ Department of Oncology, Tongji Hospital, Huazhong University of Science and Technology, Wuhan, Hubei Province, China; ^2^ Department of Statistics, Tongji Hospital, Huazhong University of Science and Technology, Wuhan, Hubei Province, China

**Keywords:** single nucleotide polymorphism, radiation-induced lung injury, lung cancer, MMP-1, radiotherapy

## Abstract

Matrix metalloproteinase-1 (MMP-1) has been implicated in several inflammatory and fibrotic diseases. We hypothesized that genetic variations in the MMP1 promoter region are associated with risk of radiation-induced lung injury (RILI). A cohort of 251 lung cancer patients was genotyped for five single nucleotide polymorphisms in the *MMP1* promoter region. We found that rs1144393 AG/GG was strongly correlated with an increased occurrence of grade ≥ 2 RILI (*p* = 0.002). Additionally, patients with the rs1144393 AG/GG genotypes exhibited higher MMP-1 expression than patients with the AA genotype in lung tissues (*n* = 28, *p* = 0.022) and plasma samples (*n* = 40, *p* = 0.018). Our results indicated that rs1144393 in the *MMP1* promoter region can be a predictor of grade ≥ 2 RILI in lung cancer patients treated with thoracic radiation.

## INTRODUCTION

Radiotherapy is an important therapeutic modality for lung cancer [1]; however, radiation-induced lung injury (RILI) diminishes the efficacy of radiotherapy [2]. Approximately 16% to 40% of lung cancer patients develop severe RILI after thoracic irradiation [3]. Therefore, establishing reliable predictors of RILI could help maximize the efficacy and minimize the adverse effects of radiotherapy. Previous studies investigated and identified multiple therapeutic and patient-related factors, such as Karnofsky performance status (KPS), chronic lung disease, smoking status, and chemotherapy, that may influence RILI risk [4–7]. Researches also demonstrated that dosimetric parameters, including mean lung dose (MLD) and percent lung volume receiving more than a threshold radiation dose (V_Dose_), might provide a guide to assess the risk of RILI in the treatment- planning process [8–10]. Nevertheless, only a small proportion of patients develop RILI when exposed to similar doses and volumes of irradiation relative to those not afflicted, suggesting that genetic factors may play a crucial role in RILI development.

Matrix metalloproteinase-1 (MMP-1) is a member of the matrix metalloproteinase family and specifically degrades collagen types I and III, which are resistant to most proteinases [11]. MMP-1 has been implicated in inflammatory and fibrotic diseases, including rheumatoid arthritis and lung emphysema [12, 13]. Intriguingly, previous research found that MMP-1 was highly upregulated in idiopathic pulmonary fibrosis (IPF), which shares similar pathological features to radiation-induced pulmonary fibrosis, the later stage of RILI [14, 15]. Moreover, studies have suggested that increased levels of circulating MMP-1 may serve as a molecular biomarker for IPF [16]. These facts suggested that MMP-1 may be involved in the RILI process.

The *MMP1* gene is located on chromosome 11q22, and its expression can be influenced by single nucleotide polymorphisms (SNPs) in its promoter region [17]. The transcriptional activation of *MMP1* is influenced by a SNP located at position −1607 (rs1799750) in the *MMP1* promoter region [18]. Specifically, other SNPs [−519 A > G (rs1144393), −422A > T (rs475007), −340 A > G (rs514921), and −320T > C (rs494379)] in the *MMP1* promoter region exhibited haplotype effects on *MMP1* activity [19]. Furthermore, several of these SNPs in the *MMP1* promoter region were associated with susceptibility to coronary artery disease, posterior tibial tendinopathy, and IPF [20–23]. To our knowledge, no studies have addressed how *MMP1*-promoter polymorphisms influence the risk of RILI. In this study, we investigated the association of these five SNPs with the occurrence of RILI in lung cancer patients treated with radiotherapy.

## RESULTS

### Patient characteristics and RILI

Clinical and pathological characteristics of the patients included in the current study are shown in Table 1. The study included 251 patients with lung cancer (164 NSCLC and 87 SCLC), 193 of whom were male and 58 were female, with 96.0% (*n* = 241) treated with a combination of chemotherapy and radiation therapy and 50.6% (*n* = 127) undergoing surgery before radiotherapy.

**Table 1:** Patient characteristics (*N* = 251)

Characteristic		No. of Patients		%
**Sex**				
	Male	193		76.9
	Female	58		23.1
**Age, years**				
	Median		58	
	Range		29–79	
**Histology**				
	SCLC	87		34.7
	NSCLC	164		65.3
**Stage**				
	I– II	25		10.0
	III–IV	226		90.0
**KPS**				
	80–100	191		76.1
	< 80	60		23.9
**Smoking**				
	Smoker	156		62.2
	Non-smoker	95		37.8
**Chemotherapy**				
	Yes	241		96.0
	No	10		4.0
**CRT**				
	Yes	67		26.7
	No	184		73.3
**Surgery**				
	Yes	127		50.6
	No	124		49.4
**IMRT**				
	Yes	153		61.0
	No	98		39.0
**Radiation dose (cGy)**				
	Median		5400	
	Range		4500–6600	
**MLD (cGy)**				
	Median		1359	
	Range		178–2017	
**V_20_**				
	Median		24.39	
	Range		0–42.00	
**COPD**				
	Yes	22		8.8
	No	229		91.2

Abbreviations: KPS, Kamofsky performance status; RT, radiotherapy; CRT, concurrent chemoradiation; IMRT, intensity-modulated radiation therapy; MLD, mean lung dose; V_20_, volume of normal lung receiving 20 Gy or more radiation; COPD, chronic obstructive pulmonary disease.

After radiotherapy treatment, 140 patients (55.8%) had grade ≥ 2 RILI (grades 2, 3, 4, and 5 were observed in 103, 32, 2, and 3 patients, respectively). Table 2 shows the associations between clinical-pathological characteristics and grade ≥ 2 RILI risk. We found that V_20_ ≥ 24% and MLD ≥ 1300 cGy were significantly associated with increased grade ≥ 2 RILI risk according to both univariate and multivariate analysis. None of the other clinical-pathological characteristics was associated with RILI risk in this study population.

**Table 2:** Association between patient characteristics and grade ≥ 2 RILI

Parameter		Univariate analysis			Mutivariate analysis		
		HR	95% CI	*P*	HR	95% CI	*P*
**Sex**							
	Male	1			1		
	Female	0.855	0.573–1.274	0.441	0.774	0.458–1.308	0.338
**Age, years**							
	< 58	1			1		
	≥ 58	1.256	0.898–1.756	0.183	1.245	0.868–1.787	0.234
**Histology**							
	SCLC	1			1		
	NSCLC	0.895	0.634–1.263	0.527	0.908	0.598–1.377	0.649
**Stage**							
	I–II	1			1		
	III–IV	0.841	0.499–1.417	0.516	0.845	0.489–1.462	0.548
**KPS**							
	80–100	1			1		
	< 80	1.280	0.878–1.864	0.199	1.342	0.906–1.989	0.143
**Smoking**							
	Smoker	1			1		
	Nonsmoker	1.062	0.753–1.497	0.733	1.116	0.706–1.764	0.637
**Surgery**							
	Yes	1			1		
	No	0.979	0.702–1.365	0.901	0.858	0.564–1.304	0.473
**Chemotherapy**							
	Yes	1			1		
	No	1.626	0.760–3.479	0.211	1.821	1.137–4.008	0.136
**CRT**							
	Yes	1			1		
	No	0.911	0.621–1.336	0.633	1.089	0.722–1.762	0.685
**IMRT**							
	Yes	1			1		
	No	1.295	0.924–1.815	0.133	1.087	0.700–1.641	0.710
**Radiation dose, cGy**							
	< 5400	1			1		
	≥ 5400	1.228	0.874–1.726	0.236	1.274	0.893–1.817	0.182
MLD cGy*							
	< 1300	1			1		
	≥ 1300	1.442	1.025–2.029	0.036	1.700	1.079–2.677	0.022
**V_20_**							
	< 24%	1			1		
	≥ 24%	1.464	1.040–2.059	0.029	1.652	1.140–2.396	0.008
**COPD**							
	Yes	1			1		
	No	1.033	0.571–1.867	0.915	0.972	0.523–1.806	0.928

Note: Multivariate analyses were adjusted for all factors in Table 1.

Abbreviations: HR, hazard ratio; KPS, Kamofsky performance status; RT, radiotherapy; CRT, concurrent chemoradiation; IMRT, intensity-modulated radiation therapy; MLD, mean lung dose; V_20_, volume of normal lung receiving 20 Gy or more radiation; COPD, chronic obstructive pulmonary disease.

* Either MLD or V_20_ was used in multivariate analyses, but not together.

### RILI and *MMP1* promoter polymorphisms

**Figure 1:**
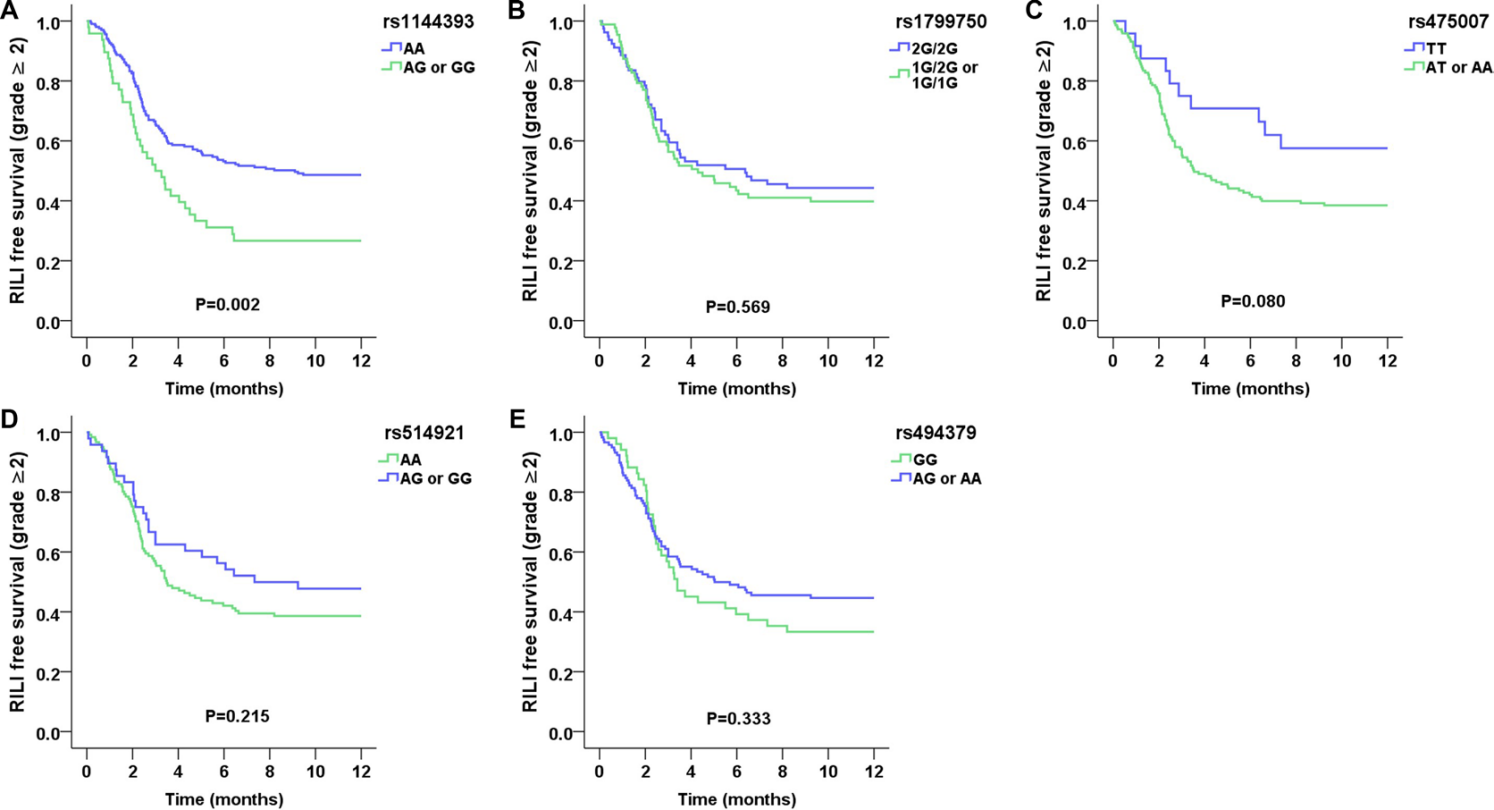
Kaplan–Meier estimates free survival of grade ≥ 2 RILI as a function of time from the start of radiation therapy by genotypes (**A**) *MMP1*:rs1144393; (**B**) *MMP1*:rs1799750; (**C**) *MMP1*:rs475007; (**D**) *MMP1*:rs514921; and (**E**) *MMP1*:rs494379. The rs1144393 AA genotype was associated with a significantly lower risk of RILI as compared with other genotypes (*p* = 0.002).

Table 3 shows the results of univariate and multivariate analyses of the associations between genetic polymorphisms and grade ≥ 2 RILI using the Cox proportional hazards model. We found that rs1144393 was significantly associated with risk of grade ≥ 2 RILI. Compared with the rs1144393 AA genotype, the GG/AG genotypes were associated with increased risk of grade ≥ 2 RILI (hazard ratio = 1.821; 95% CI, 1.241–2.674; *p* = 0.002). Similar results were observed in multivariate analyses with adjustment for potential confounding factors of RILI, including age, sex, race, KPS, disease stage, tumor histology, smoking history, use of chemotherapy, radiation dose, and V_20_. Figure 1A plots the incidence of grade ≥ 2 RILI as a function of time after radiation therapy according to the presence of rs1144393. Development of grade ≥ 2 RILI was prolonged and the incidence remained higher in the rs1144393 AG/GG genotypes, while no associations with grade ≥ 2 RILI were found for the other SNPs (Figure 1B–1E).

**Table 3:** Association between MMP1 genotypes and Grade ≥ 2 RILI

Polymorphism and Genotype	No.of event	No.of total	Univariate analysis			Multivariate analysis		
			HR	95% CL	*P*	HR	95% CL	*P*
*MMP1:rs1799750*								
2G/2G	44	79	1			1		
1G/2G	42	69	1.156	0.757–1.765	0.502	1.175	0.755–1.830	0.474
1G/1G	10	18	1.004	0.505–1.995	0.992	0.970	0.474–1.985	0.934
1G/2G	52	87	1.123	0.752–1.679	0.571	1.129	0.743–1.715	0.571
+1G/1G								
*MMP1:rs1144393*								
AA	104	203	1			1		
AG	36	47	1.877	1.279–2.756	**0.001**	1.858	1.249–2.763	0.010*
GG	0	1	NC			NC		
AG + GG	36	48	1.821	1.241–2.674	**0.002**	1.805	1.211–2.688	0.020*
*MMP1:rs475007*								
TT	53	81	1			1		
AT	36	64	0.796	0.521–1.217	0.292	0.889	0.565–1.379	0.609
AA	10	24	0.496	0.252–0.976	0.042	0.555	0.270–1.140	0.109
AT + AA	46	88	0.704	0.474–1.046	0.082	0.796	0.518–1.225	0.300
*MMP1:rs514921*								
AA	74	121	1			1		
AG	24	54	0.779	0.491–1.235	0.288	0.803	0.493–1309	0.380
GG	1	3	0.408	0.057–2.939	0.374	1.049	0.132–8.315	0.964
AG + GG	25	48	0.752	0.477–1.184	0.218	0.811	0.501–1.313	0.393
*MMP1:rs494379*								
GG	34	51	1			1		
AG	46	80	0.880	0.565–1.371	0.572	0.841	0.518–1.366	0.485
AA	19	38	0.692	0.394–1.214	0.199	0.784	0.432–1.420	0.422
AG + AA	65	118	0.815	0.538–1.235	0.335	0.823	0.523–1.296	0.400

NOTE: Multiple analyses in this table were adjusted for all the factors listed in Table 1.

Abbreviations: MMP-1, Matrix Metalloproteinase-1; HR, hazard ratio.

*NC* not calculated.

* *P* value were adjusted by Bonferroni corrections.

### RILI and MMP-1 expression

**Figure 2:**
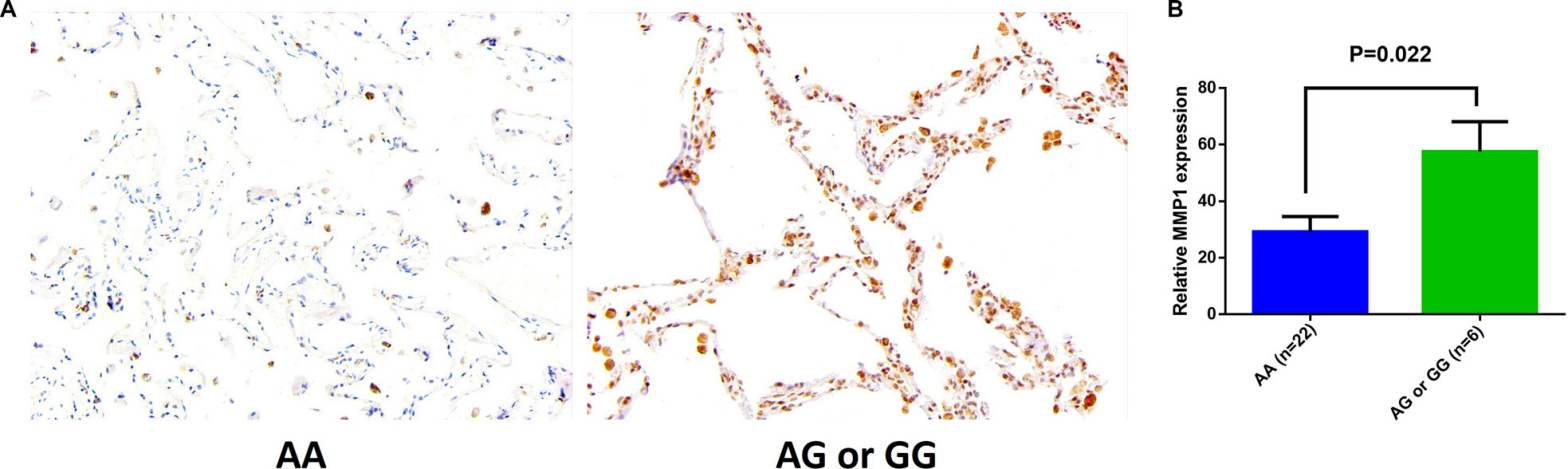
Immunohistochemical (IHC) staining for MMP-1 in normal lung tissues (**A**) Representative photomicrograph of pneumocytes with rs1144393 AA or AG/GG genotypes. Original magnification, 20×. (**B**) IHC quantification data for MMP-1 expression in lung tissues from patients with the rs1144393 AA genotype (*n* = 22) and the rs1144393 AG or GG genotypes (*n* = 6) were evaluated by the mean optical density per pixel obtained from alveolar areas. Significant differences were identified using the Mann–Whitney *U* test (*p* = 0.022), data are represented as mean ± SEM.

**Figure 3:**
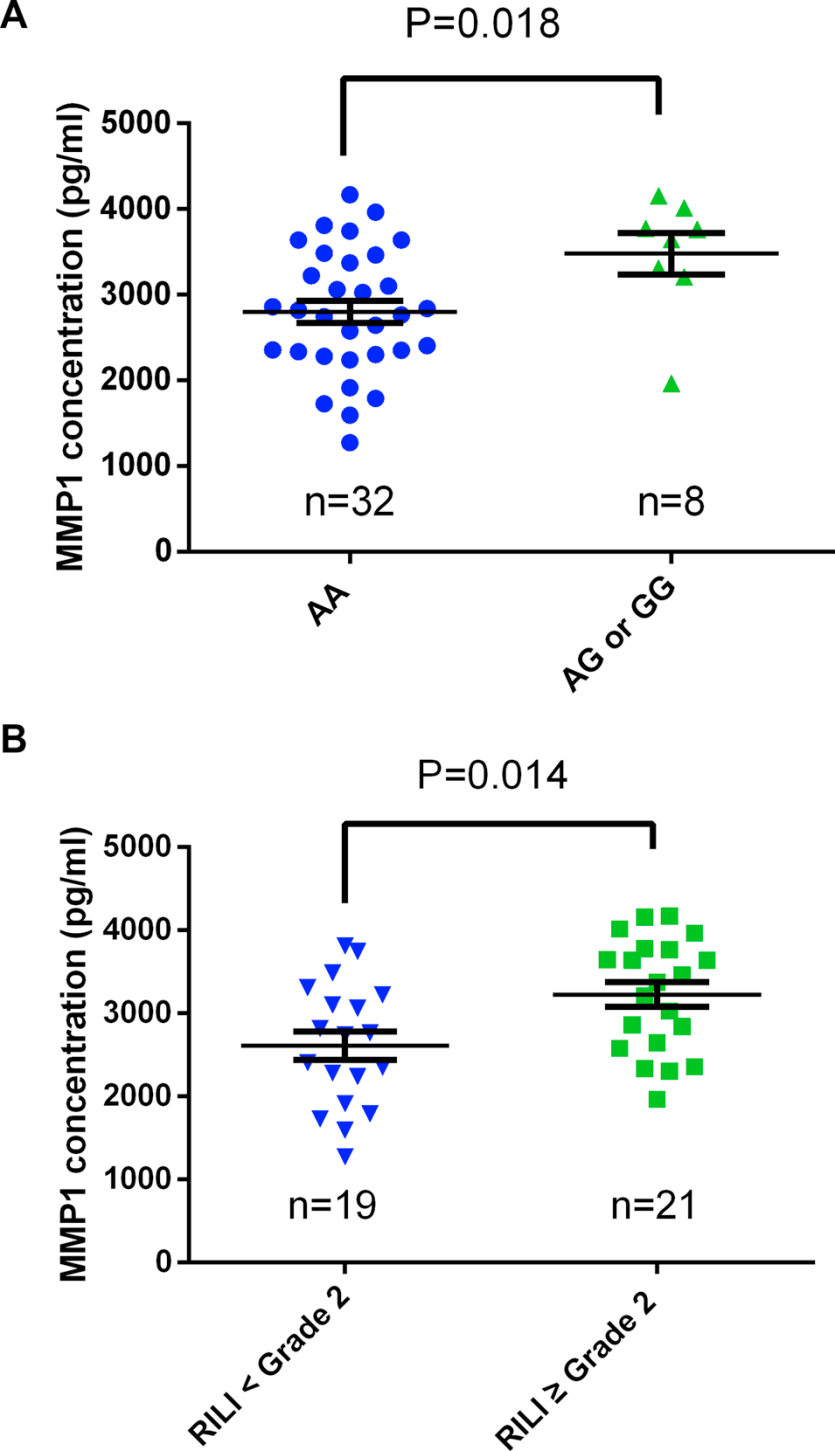
Plasma MMP-1 concentrations in lung cancer patients (**A**) Comparison of plasma MMP-1 concentrations between patients with the rs1144393 AA genotype (*n* = 32) and those with the AG or GG genotypes (*n* = 8; *p =* 0.018). (**B**) Comparison of plasma MMP-1 levels between patients displaying grade < 2 RILI (*n* = 19) and those displaying grade ≥ 2 RILI (*n* = 21; *p* = 0.014). Significant differences were determined using the Mann–Whitney *U* test, data are represented as mean ± SEM.

To investigate the functional impact of rs1144393 on MMP-1, we detected MMP-1 expression in patients with different rs1144393 genotypes by immunohistochemical staining. Compared to patients with the AA genotype, patients with unfavorable AG/GG genotypes exhibited higher MMP-1 expression levels in lung tissues (*n* = 28, *p* = 0.022; Figure 2). Moreover, we examined the plasma MMP-1 concentration in samples collected 3–6 months after completion of radiotherapy by 40 patients from our cohort. Similar to immunohistochemical results, patients with AG/GG genotypes exhibited higher MMP-1 concentrations (*n* = 40, *p* = 0.018; Figure 3A, Supplementary Table S1). Additionally, we found that MMP-1 concentrations in patients currently or subsequently displaying grade ≥ 2 RILI were significantly higher relative to those with lower-grade RILI (*p* = 0.014; Figure 3B). We also observed that MMP-1 concentration was associated with V_20_ (Supplementary Figure S1).

## DISCUSSION

Here, we tested whether genetic polymorphisms in the *MMP1* promoter region might be associated with RILI risk in lung cancer patients treated with radiotherapy. To our knowledge, this is the first report of an association between the presence of rs1144393 and occurrence of grade ≥ 2 RILI. Patients with rs1144393 AG or GG genotypes exhibited increased risk of RILI following radiotherapy. Moreover, patients with rs1144393 AG/GG genotypes displayed higher MMP-1 expression levels in tissue and plasma samples relative to patients with the AA genotype.

It was reported that polymorphisms in the *MMP1* promoter may alter gene expression. The rs1144393 polymorphism is located in this region and is characterized by the substitution of an adenine (A) with a guanine (G). Previous reports indicated that rs1144393 is a risk factor for coronary artery disease [24], and it was associated with hypertension and intima-media thickness [25]. Additionally, the G-to-A substitution in rs1144393 resulted in a greater risk of posterior tibial tendinopathy [22]. These findings suggested that this polymorphism is functional in inflammatory or fibrotic diseases, including RILI. We also investigated the functional impact of rs1144393 on MMP-1 expression in lung cancer patients and found that patients with rs1144393 AG/GG genotypes exhibited higher MMP-1 expression in lung tissues and plasma samples relative to patients with the AA genotype. This finding was consistent with a study reporting that haplotypes of this polymorphism increased promoter activity and altered MMP-1 expression [26]. However, in this study, we did not observe associations between other polymorphisms in the *MMP1* promoter region and RILI. This includes rs1799750, which confers increased risk for IPF and could influence transcription and translation of *MMP1* [23]. This may have been due to the different natures of the diseases and the small sample size of the population.

In this study, we also found that the mean plasma MMP-1 concentration in post-treatment plasma samples from patients with grade ≥ 2 RILI was higher relative to that observed in patients with lower-grade RILI. Although little is known about the association of MMP-1 concentrations with RILI, studies have found that increased levels of circulating MMP-1 and MMP-7 may serve as molecular biomarkers for IPF [16]. MMP-1 serum levels were significantly higher in IPF as compared to non-IPF usual interstitial pneumonia [27]. These findings implied that MMP-1 has similar functions in IPF and RILI. Moreover, we observed that MMP-1 concentration was associated with dosimetric parameters. This is consistent with other reports that infrared or ultraviolet irradiation could dose-dependently induce MMP-1 expression [28–32]. These findings indicate that an association between MMP-1 expression and RILI is biologically plausible. However, because we did not have plasma samples prior to radiotherapy, we could not determine whether this effect was induced by radiotherapy or due to other factors. Further investigations involving serial blood samples collected at different time points prior to and after radiotherapy are required to validate this finding.

Our study suggested that the rs1144393 SNP located in the *MMP1* promoter region can be used as a predictor of RILI risk before radiotherapy in addition to radiation dosimetric parameters. In combination with our previous findings concerning RILI-susceptibility SNPs in *TGFβ1, ITGB6, PI3CA* and *AKT2* [33–35], we are able to establish a more accurate model using these variants, enabling patients to benefit from early prediction and prevention of RILI by genotyping prior to radiotherapy. This would enable patients lacking RILI-susceptibility genotypes to receive appropriately elevated radiation doses to enhance tumor-related therapies. Moreover, our results concerning higher MMP-1 plasma concentrations in patients with grade ≥ 2 RILI suggested a critical role of MMP-1 in the onset of RILI, which may aid the discovery of targets to treat RILI.

Despite these positive findings, our current study had some limitations. First, we did not discover an association between *MMP1* promoter polymorphisms and grade ≥ 3 RILI. Since grade ≥ 3 RILI is more likely to result in clinical consequences, our findings need to be confirmed by studies on a larger sample size in order to validate this association. Additionally, we were unable to determine the exact molecular mechanisms by which *MMP1* promoter polymorphisms lead to RILI in patients with lung cancer. Finally, since some genetic variants are ethnicity-specific, our results should be validated in different races in the future.

In conclusion, this study identified that rs1144393 AG/GG genotypes located in the *MMP1* promoter region were associated with increased RILI risk in patients with lung cancer treated with radiotherapy. Our findings suggested that this polymorphism could be used as a predictive factor of RILI in patients with lung cancer prior to initiating radiation therapy. However, larger prospective studies are needed to validate these findings.

## MATERIALS AND METHODS

### Patients

**Figure 4:**
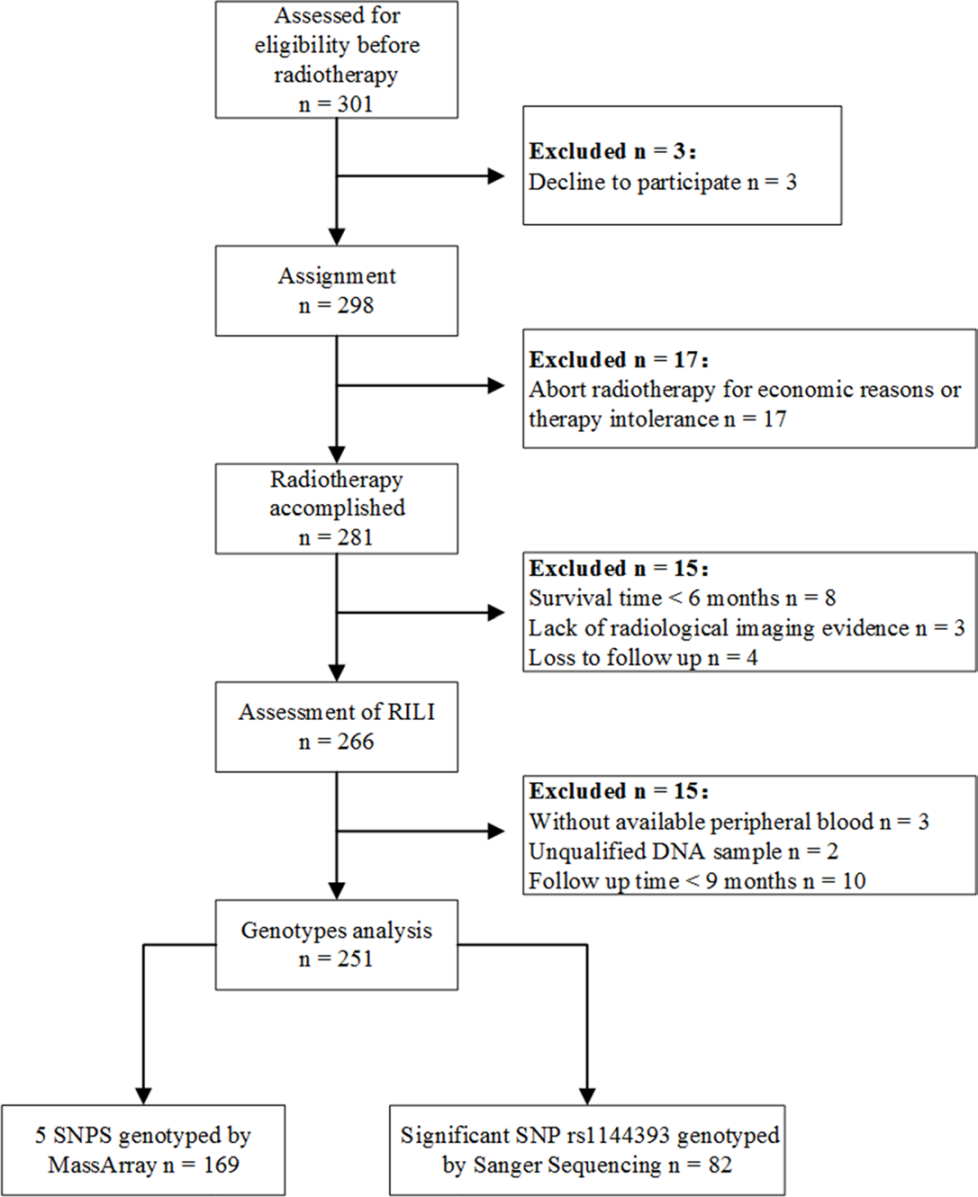
Patient flow diagram

For this prospective study (NCT02490319), 301 lung cancer patients were initially enrolled. All patients were treated with radiation therapy at Tongji Hospital, Huazhong University of Science and Technology (Wuhan, Hubei Province, China), between 2008 and 2015. Among the initially enrolled patients, 251 who had received a radiation dose ≥ 45 Gy (KPS > 60) and had a life expectancy of ≥ 6 months and a ≥ 9-month follow-up time were finally enrolled in our study (Figure 4). Patients with previous thoracic irradiation or severe cardiopulmonary diseases were excluded. Written informed consent was obtained from all patients for access to their clinical information and DNA. This study was approved by the Review Board of Tongji Hospital. Samples from 169 patients (September 2008 to June 2014) were first used to genotype the five candidate SNPs by MassArray in order to screen for RILI-susceptibility variants. The significantly associated SNPs were then genotyped by Sanger sequencing in the remaining 82 patients (June 2014 to February 2015).

### Treatment and follow-up

All patients received radiotherapy with 6-MV X-rays from a linear accelerator (Elekta Synergy, Elekta, Sweden). The median total radiation dose was 54 Gy (range: 45–66 Gy), with 1.5–2 Gy administered per radiation treatment. Intensity-modulated radiation therapy (IMRT) was administered to 61% of the patients (*n* = 153), and computed tomography simulation (CT/e; GE, Fairfield, Connecticut, USA) was performed before radiation therapy treatment was planned. The target volumes and critical normal organs were delineated by the three-dimensional planning system (Pinnacle Version 9.2; Philips Healthcare, Amsterdam, Netherlands).

All patients included in this study were examined during treatment and at 1 month post-treatment. The patients were followed up with every 3 months for the first year and every 6 months thereafter. Radiographic examination by chest X-ray or CT was performed at each follow-up visit after completion of treatment. RILI was graded by two radiation oncologists according to the Common Terminology Criteria for Adverse Events 4.0 as follows: grade 0, no change; grade 1, asymptomatic and diagnosed by radiographic findings only; grade 2, symptomatic, medical intervention indicated, not interfering with daily activities; grade 3, symptomatic, interfering with daily activities or oxygen required; grade 4, assisted ventilation required; grade 5, fatal.

### Genotyping methods

Genomic DNA from 251 patients was extracted from peripheral blood with a PureLink Genomic DNA Mini Kit (K1820-01; Invitrogen, Carlsbad, CA, USA) according to the manufacturer's instructions. Five SNPs in the *MMP1* promoter region were selected for analysis (rs1799750, rs1144393, rs475007, rs514921, and rs494379). All SNPs were identified with a cut-off value of *r*^2^ > 0.8 and a minor allele frequency > 0.1 in the Chinese population based on HapMap HCB data. Genotypes were determined using the MassArray system (Sequenom iPLEX^®^ assay, Sequenom, Inc., San Diego, CA, USA) for the first 169 patients. Sample DNA was amplified by a multiplex PCR reaction, and the PCR products were then used for a locus-specific single-base extension reaction. The resulting products were desalted and transferred to a 384-element SpectroCHIP array (Sequenom). The alleles were discriminated by mass spectrometry (Sequenom). The RILI-susceptibility SNP rs1144393 was then genotyped in the remaining 82 patients by Sanger sequencing using the following primers: forward (5′-CCCCAgCACTCACTTTACgg-3′) and reverse (5′-gCAAggggTggggAgTTATC-3′). The PCR products were then subjected to DNA sequencing to detect mutations.

### Immunohistochemical analysis

Tumor-adjacent lung tissues were obtained from 28 surgery patients in our cohort prior to radiotherapy, and then fixed in 10% formalin overnight, paraffin-embedded, and sectioned at an average thickness of 4 μm. Deparaffinized sections were subjected to antigen retrieval and then incubated with 3% hydrogen peroxide for 15 min. After blocking with 5% bovine serum albumin, the sections were incubated with anti-MMP-1 antibody (AP11874c,1:50, Abgent, San Diego, CA, USA) overnight at 4°C, followed by incubation with secondary goat anti-rabbit antibody (GB23303; Wuhan Goodbio Technology Co., Wuhan, China) for 50 min. Counterstaining with hematoxylin was performed, and 3,3′-diaminobenzidine positivity was analyzed. Images were captured using an Eclipse TE2000-S microscope (Nikon, Tokyo, Japan). The mean optical density obtained from alveolar areas for each stained slide was quantified by Image-Pro Plus 6.0 analysis software (Media Cybernetics, Rockville, MD, USA).

### Enzyme-linked immunosorbent assay (ELISA)

Plasma samples from 40 patients in our cohort were tested by ELISA. These samples were collected during the same period (3–6 months) following completion of radiotherapy. All plasma samples were harvested with pyrogen-free heparinized tubes, immediately placed on ice for 30 min, and then spun at 3000 rpm for 15 min at 4°C to separate the plasma. Plasma samples were immediately aliquoted and stored at −80°C for future use. Plasma MMP-1 concentrations were measured by ELISA (Elabscience Biotechnology Co., Wuhan, China) according to the manufacturer's instructions. Concentrations were estimated from a standard curve and expressed as pg/mL, with the minimum detectable dose determined at 94 pg/mL.

### Statistical analysis

The end-point for this study was the time for developing grade ≥ 2 RILI, which was calculated from the start of radiotherapy. The data from patients who did not experience grade ≥ 2 RILI within 12 months of radiotherapy were censored. Statistical analysis was performed using IBM SPSS 19.0 (IBM, Corp., Armonk, NY, USA). Hazard ratios with 95% confidence intervals (CIs) of genotypes for RILI were computed by the Cox proportional hazards model. Multivariate Cox regression analysis was used for the adjustment of covariates. Moreover, the influences of the genotypes on RILI risk were assessed by Kaplan–Meier analysis and compared with log-rank tests. The Mann–Whitney *U* test was used to analyze the data obtained from ELISA and immunohistochemistry. Values of *p* ≤ 0.05 were considered statistically significant.

## SUPPLEMENTARY MATERIALS


