# Supplementary material for: MMP-1 promoter polymorphism is associated with risk of radiation-induced lung injury in lung cancer patients treated with radiotherapy
Source: Oncotarget. 2016 Sep 21;7(43):70175–84. doi: 10.18632/oncotarget.12164 (PMC5342544; doi:10.18632/oncotarget.12164)
Supplement: Supplementary file 1 [file oncotarget-07-70175-s001.pdf]

MMP-1 promoter polymorphism is associated with risk of radiation-induced lung injury in lung cancer patients treated with radiotherapy

Supplementary Materials

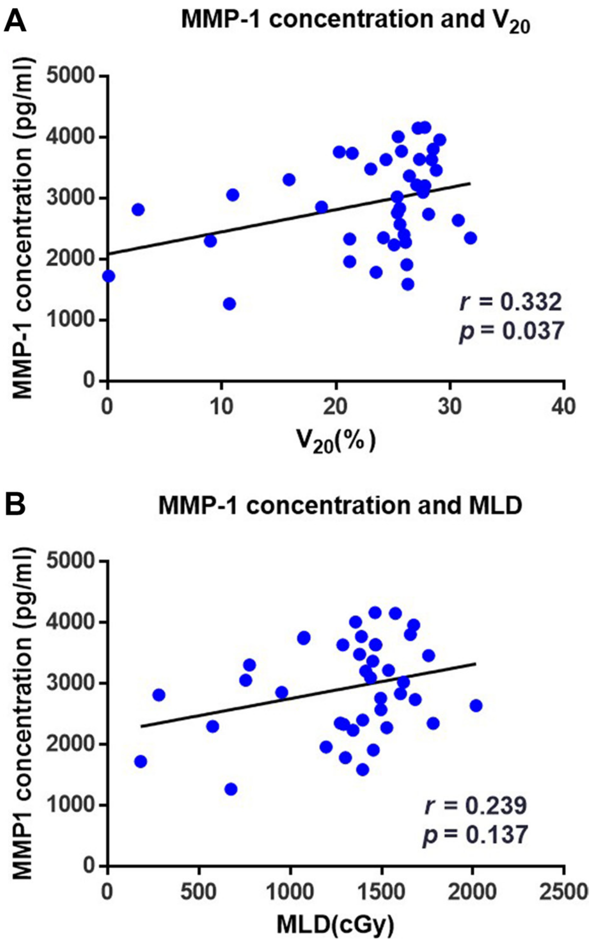

Supplementary Figure S1: Correlation between lung dosimetric parameters and MMP-1 concentration. Spearman correlation coefficients were calculated for linear relations between lung dosimetric parameters and MMP-1 concentration (**A**) V_{20} and MMP-1 concentration. (**B**) Mean lung dose (MLD) and MMP-1 concentration.

Supplementary Table S1: *MMP1* genotypes and MMP-1 concentration

MMP1 genotypes	MMP-1 concentration (pg/ml)	*P*
MMP1:rs1799750		
2G/2G	3286.56 ± 185.58	**0.022**
1G/2G + 1G/1G	2688.32 ± 148.46	
MMP1:rs1144393		
AA	2797.28 ± 129.42	**0.018**
AG + GG	3480.29 ± 244.10	
MMP1:rs475007		
TT	2721.04 ± 414.54	0.649
AT + AA	2971.45 ± 124.75	
MMP1:rs514921		
AA	2902.75 ± 139.52	0.617
AG + GG	3027.29 ± 255.74	
MMP1:rs494379		
GG	2770.10 ± 189.56	0.223
AG + AA	3054.94 ± 156.36	

NOTE: *P* values were calculated by the Mann–Whitney *U* test. Data are mean ± SEM.
